# A Multidimensional Assessment of Cardiometabolic Risk in Asian Americans with Chronic Hepatitis B Using the All of Us Datasets

**DOI:** 10.3390/jcm15197371

**Published:** 2026-09-23

**Authors:** Lin Zhu, Amanda C. Yang, Han Le, Lena Dong, Aisha Bhimla, Wenyue Lu, Xiaoli Ma, Kaizhe Zhang, Olivia Gong, Chun Pan, Grace X. Ma

**Affiliations:** 1 Center for Asian Health, Department of Population Health Sciences, Lewis Katz School of Medicine, Temple University, 3440 N. Broad St., Philadelphia, PA 19140, USA; amanda.yang18@temple.edu (A.C.Y.); lena.dong@temple.edu (L.D.); aisha.bhimla@temple.edu (A.B.); wenyue.lu@temple.edu (W.L.); olivia.gong@temple.edu (O.G.); grace.ma@temple.edu (G.X.M.); 2 Hepatology Clinic, 121 N. 9th St., Philadelphia, PA 19107, USA; tangmali@yahoo.com; 3 Department of Sociology, College of Arts & Science, New York University, 79 Washington Square East, New York, NY 10003, USA; 4 Department of Mathematics and Statistics, Hunter College, The City University of New York, 695 Park Avenue, New York, NY 10065, USA; cp2677@hunter.cuny.edu

**Keywords:** chronic hepatitis B, cardiometabolic conditions, comorbidity, Asian American, chronic disease, whole person health

## Abstract

**Background/Objectives**: Asian Americans (AAs) bear a disproportionate burden of chronic hepatitis B (CHB), with one in 12 living with this condition. Despite evidence that cardiometabolic conditions accelerate CHB progression and hepatocellular carcinoma risk, cardiometabolic comorbidities in this population remain critically understudied. This study provides an assessment of cardiometabolic burden and phenotypic heterogeneity among AAs with CHB. **Methods**: We analyzed data from the All of Us Research Program Controlled Tier Dataset v8 (*n* = 436 AAs with CHB). We ran descriptive analyses to examine four cardiometabolic conditions (hypertension, type 2 diabetes mellitus [T2DM], dyslipidemia, and central obesity). Unsupervised clustering (k-prototypes and k-modes) identified cardiometabolic phenotypes in the overall sample and stratified by sex and age. Multinomial logistic regression examined sociodemographic predictors of cluster membership. **Results**: Cardiometabolic burden was substantial: 38.76% had hypertension, 25.46% dyslipidemia, 17.66% T2DM, and 16.74% central obesity. Nearly two-thirds (61.93%) had at least one cardiometabolic condition, and over one-quarter (28.90%) had two or more. Cluster analysis identified three phenotypes: “Young/Healthy” (54.6%), “Dyslipidemia-Dominant” (19.7%), and “Hypertension-Dominant” (25.7%). Sex- and age-stratified analyses revealed divergent metabolic profiles not apparent in the overall solution. **Conclusions**: AAs with CHB carry a high and heterogeneous cardiometabolic burden. Integrated metabolic screening and sex- and age-informed prevention strategies may be warranted for this high-risk population, though prospective validation is needed to establish the clinical utility of these phenotypes.

## 1. Introduction

Chronic hepatitis B (CHB) affects approximately 2.2 million individuals in the U.S. and remains a major cause of hepatocellular carcinoma (HCC) [1,2]. Asian Americans (AAs) bear a disproportionate burden, with one in 12 living with CHB [1,3]. Emerging evidence indicates that cardiometabolic comorbidities substantially influence CHB disease trajectories [4,5]. Specifically, growing evidence has suggested that cardiometabolic conditions such as central obesity, hypertension, dyslipidemia, and hyperglycemia [6] accelerate liver fibrosis progression, impair antiviral treatment response, and increase HCC risk in CHB patients [7,8,9,10]. Of particular concern, metabolic dysfunction-associated steatotic liver disease (MASLD), the hepatic manifestation of cardiometabolic burden, defined by hepatic steatosis in the absence of excessive alcohol consumption [11], is increasingly prevalent globally [12,13] with disproportionately high rates reported among individuals of Asian descent both within Asia and in diaspora populations [14,15]. Given that AAs experience the highest CHB burden in the U.S. and that cardiometabolic–viral interactions critically impact clinical outcomes, characterizing the cardiometabolic disease landscape in this population would be valuable for informing screening and prevention strategies.

Traditional epidemiological approaches examine cardiometabolic conditions in isolation, assessing hypertension, diabetes, or dyslipidemia as independent risk factors [16,17,18] or employ binary metabolic syndrome (MetS) classification (≥3 of 5 components) [19,20]. However, these methods obscure critical heterogeneity. Individuals classified as having MetS may present vastly different cardiometabolic profiles: some exhibit obesity-driven glucose dysregulation, others lipid-dominant patterns without significant weight gain, and still others hypertension-focused phenotypes [21,22]. These distinct phenotypes carry different prognostic implications [21,22]. In the context of CHB, this heterogeneity remains uncharacterized, limiting our ability to tailor cardiometabolic screening and management strategies.

Phenotyping approaches using unsupervised clustering methods address these limitations by identifying naturally occurring subgroups based on multidimensional cardiometabolic profiles rather than arbitrary cut-off points. This approach is particularly valuable for Asian American populations, who remain understudied despite exhibiting distinct cardiometabolic characteristics, including cardiometabolic comorbidities at lower BMI thresholds [23,24,25] and visceral adiposity accumulation without generalized obesity [26,27]. Among individuals with CHB, characterizing cardiometabolic profiles could illuminate which cardiometabolic patterns most strongly interact with viral liver disease and guide precision prevention strategies. Despite the clinical relevance, to our knowledge, no studies have applied phenotyping methods to characterize cardiometabolic heterogeneity in a national sample of Asian Americans living with CHB.

## 2. Materials and Methods

We used data from All of Us (AoU), an ongoing cohort study that was launched nationally in May 2018 with funding from the National Institutes of Health [28]. Open for enrollment in 2018, AoU provides unprecedented datasets for researchers to reduce disparities and enable more precise approaches to care [29]. So far, more than 883,000 individuals have enrolled in the program [30]. Data for this study (v8) were collected between May 2018 and October 2023. This version included approximately 633,000+ survey responses, 509,000+ physical measurements, 393,000+ electronic health records (EHRs), and other data on genotyping arrays, whole genome sequences, and Fitbit records [31]. Eligibility criteria for enrollment include adults (18 years or older) living in the United States. 

The AoU Research Program is a national prospective cohort that collects multimodal data from enrolled participants, including online health surveys, physical measurements, EHR data, and biological biospecimens (blood, urine, or saliva). Participants may enroll through the All of Us website or mobile application and can do so either on-site via a health care provider organization (HPO), such as regional medical centers or federally qualified health centers, or remotely through a “direct volunteer” (DV) pathway for those not affiliated with a participating health center [32]. Following informed consent, participants complete three baseline surveys assessing sociodemographic characteristics, general health (including health literacy, quality of life, and physical and mental health status), and health behaviors (including tobacco, alcohol, and substance use). Physical measurements and biospecimen collection occur either at an HPO site or via the DV mechanism. Enrollment materials and surveys are currently available in English and Spanish only. A full description of the study design and research protocol has been published elsewhere [28].

Using the Cohort Builder in the Researcher Workbench, we identified 22,400 adult Asian Americans (self-identified) in the AoU v8 database, among which 11,690 had EHR data. Among the 11,690 participants, we then identified 436 who had chronic hepatitis B. AoU participants provided consent to participate in the study, the protocol of which was approved and is actively monitored by the All of Us Institutional Review Board [28]. Results are reported in compliance with the AoU Data and Statistics Dissemination Policy, which restricts group counts under 20 to protect participant privacy. All data from AoU were de-identified; therefore, the requirement for Institutional Review Board review was waived for this study.

Chronic hepatitis B status was ascertained by combining data from two AoU domains. Participants were classified as having CHB if they responded “yes” to the survey item “Have you ever been diagnosed with Hepatitis B?” or if their EHR-linked conditions data included a diagnosis of “chronic hepatitis B” (i.e., a positive response or documented diagnosis in either domain was sufficient for classification). Specifically, EHR-based CHB status was ascertained from the AoU Condition domain, which captures documented disease diagnoses based on ICD-9 and ICD-10 codes mapped to standard clinical vocabularies, including the Systematized Nomenclature of Medicine (SNOMED) [33]. Of the 436 participants included in the analysis, 359 (82.3%) had EHR records consistent with chronic hepatitis B infection, while the remaining 77 had no confirmed CHB diagnosis in their EHR, likely reflecting care received outside health systems linked to the All of Us program rather than diagnostic discordance. Nine (9) participants had EHR-confirmed CHB status but did not self-report their diagnosis, potentially reflecting stigma-related underreporting or survey response error. We excluded patients with excessive alcohol consumption (≥4 drinks/week), coinfected with other hepatotropic viruses or with human immunodeficiency virus (HIV), and those with a history of HCC.

Four cardiometabolic comorbidities were examined in this study: central obesity, type 2 diabetes mellitus (T2DM), hypertension, and dyslipidemia. Central obesity was defined as waist circumference ≥90 cm for men or ≥80 cm for women, using data from the AoU physical measurements domain. Type 2 diabetes was ascertained by combining self-reported survey data (“Have you ever been diagnosed with type 2 diabetes?”) with EHR-derived diagnoses from the Condition domain with International Classification of Diseases (ICD) codes. Participants were classified as having T2DM if they reported a diagnosis, had a documented T2DM diagnosis in their EHR, or reported current use of antidiabetic medication. Hypertension was similarly ascertained using self-reported survey data (“Have you ever been diagnosed with high blood pressure [hypertension]?”) and EHR-derived diagnoses from the Condition domain. Participants were classified as having hypertension if they reported a diagnosis, had a documented hypertension diagnosis in their EHR, or reported current use of antihypertensive medication. Dyslipidemia was defined using self-reported survey data (“Have you ever been diagnosed with high cholesterol?”) and EHR-derived diagnoses from the Condition domain. Participants were classified as having dyslipidemia if they met either criterion (positive self-report or documented diagnosis).

Two modifiable lifestyle behaviors were examined: tobacco use and alcohol use. Tobacco use was defined as reporting at least one of the following: having smoked 100 or more cigarettes in one’s lifetime, currently using an electronic nicotine product some days or every day, or currently smoking traditional cigars some days or every day. Alcohol use was defined as consuming alcohol two to three times per week or more frequently in the past year. Demographic and socioeconomic characteristics were derived from self-reported data in the AoU survey modules and included age group (18–44, 45–64, 65+ years), biological sex at birth (female, male), marital status (married/domestic partner vs. separated/divorced/widowed), nativity status (foreign-born vs. U.S.-born), educational attainment (less than a college degree vs. college degree or above), and annual household income (<$50,000 vs. ≥$50,000).

Descriptive statistics (frequencies and percentages) were used to summarize sociodemographic characteristics, lifestyle behaviors, and cardiometabolic comorbidities among Asian American participants with CHB. Missing data were present for waist circumference (*n* = 43, 9.9%), reflecting participants without physical measurements, and for EHR-derived conditions (*n* = 19, 4.4%), reflecting participants without linked EHR records. For participants missing waist circumference data, central obesity was classified using self-reported obesity status as a proxy. Sociodemographic variables with missing values (sex, education, income, and nativity, each missing in <10% of the analytic sample) were handled with simple imputation prior to analysis. The final analytic sample was 436.

We explored cardiometabolic phenotypes using unsupervised clustering methods. For the overall sample and sex-stratified analyses (males and females separately), we implemented k-prototype clustering using the kproto() function (*clustMixType* package, version 0.5-2 in R) with 25 random starts (nstart = 25), retaining the solution with the lowest total within-cluster sum of squares. A random seed was set prior to all analyses to ensure reproducibility (set.seed(123)). Default variable weighting was applied, balancing Euclidean distance for continuous variables (age) and simple matching distance for categorical variables. The optimal number of clusters was evaluated across k = 2 to k = 5 using within-cluster sum-of-squares plots; as the plots showed a gradual rather than sharp decline without a definitive elbow, the three-cluster solution was selected based on diminishing marginal variance reduction supplemented by clinical interpretability criteria assessing phenotype distinctiveness, cluster size, and consistency with known cardiometabolic patterns in Asian American populations. Age was included as a continuous variable (in years) in the overall and sex-stratified clustering models. For age-stratified analyses (<65 years and ≥65 years), we used k-mode clustering (*klaR* package, version 1.7-4 in R), designed specifically for categorical data, as age could not be included as a clustering variable after stratification. Both algorithms partition observations into k distinct clusters by minimizing within-cluster variation. We determined the optimal number of clusters by examining within-cluster sum-of-squares plots (for k-prototypes) and evaluating clinical interpretability. We intentionally restricted clustering variables to binary cardiometabolic conditions and age. Lifestyle behaviors (tobacco use and alcohol consumption) were excluded from clustering models and examined separately as behavioral correlates of cluster membership to avoid circularity whereby behavioral variables simultaneously define and are associated with phenotypes. We conducted cluster analysis for the overall study sample as well as stratified by biological sex (male, female) and age group (<65, ≥65 years) to examine potential differences in cardiometabolic phenotypes across demographic subgroups. All analyses were conducted using the open-source R version 4.6.1 [34] programming language through the AoU Researcher Workbench platform supported by Google Cloud.

## 3. Results

The sociodemographic characteristics of the study population are presented in Table 1. Among the 436 AA patients living with CHB, 51.61% were female. Approximately one-fifth (20.87%) were aged 18–44 years, 42.43% were 45–64 years, and 36.70% were 65 years or older. Over two-thirds (69.95%) of participants were married or living with a domestic partner. The majority (82.34%) of participants were also foreign-born. The sample demonstrated notably high socioeconomic status: 88.99% held college degrees, and 65.14% reported annual household incomes ≥$50,000. Regarding modifiable lifestyle behaviors, only 4.59% reported tobacco use, while 10.32% were identified as frequent drinkers, i.e., consuming alcohol two to three times per week or more frequently in the past year.

As shown in Figure 1, we observed a substantial cardiometabolic disease burden among AAs with CHB. One in six (16.74%) had central obesity, and nearly one-fifth (17.66%) had T2DM. The prevalence of dyslipidemia and hypertension was even higher. One-quarter (25.46%) had dyslipidemia, and over one-third (38.76%) had hypertension. Notably, nearly two-thirds (61.93%) had at least one cardiometabolic condition, and over one-quarter of the sample had two or more cardiometabolic conditions.

K-prototypes clustering identified three distinct cardiometabolic phenotypes (Figure 2). Cluster 1 (“Young/Healthy,” *n* = 238, 54.6%) was the largest group, comprising over half the sample, with the youngest mean age (49.5 years). This cluster exhibited minimal metabolic burden across all conditions: hypertension 0.8%, type 2 diabetes 3.4%, dyslipidemia 10.5%, and central obesity 15.1%. All four metabolic condition prevalences were lowest in this cluster. Cluster 2 (“Dyslipidemia-Dominant,” *n* = 86, 19.7%) was the oldest group (mean age 69.6 years) and uniquely characterized by universal dyslipidemia (100%), a feature completely absent in Cluster 3. This cluster also showed high hypertension (77.9%) and moderately high diabetes (31.4%) prevalence, representing a multimorbid phenotype. Central obesity prevalence was 20.9%, the highest among all clusters. Cluster 3 (“Hypertension-Dominant,” *n* = 112, 25.7%) had a mean age of 66.4 years, similar to Cluster 2. This cluster was distinguished by near-universal hypertension (89.3%) and the highest diabetes prevalence (37.5%), but notably, complete absence of dyslipidemia (0%), a stark contrast to Cluster 2’s universal dyslipidemia. Central obesity prevalence was 17.0%. Across all three clusters, central obesity prevalence remained moderate (15–21%), while age and specific metabolic condition patterns clearly differentiated the phenotypes. It should be noted that extreme prevalence values (including 100% dyslipidemia in the “Dyslipidemia-Dominant” cluster and 0% in the “Hypertension-Dominant” cluster) are an inherent feature of clustering algorithms applied to binary variables and should be interpreted with caution, particularly for smaller clusters (*n* = 40–86 in subsequent stratified analyses), where estimates may be unstable. We note that readers should interpret these cluster-specific prevalence estimates with caution.

To examine whether these exploratory cardiometabolic phenotypes reflected distinct behavioral profiles, we tested associations between cluster membership and two modifiable lifestyle behaviors (tobacco use and alcohol use) using chi-square tests. No significant associations were found in the overall sample or in any of the age- or sex-stratified analyses, indicating that the identified phenotypes are defined by metabolic condition patterns rather than behavioral differences.

Additionally, we conducted multinomial logistic regression with the “Young/Healthy” cluster as the reference group to evaluate sociodemographic predictors of cluster membership. Older age was associated with a significantly higher likelihood of membership in the “Dyslipidemia-Dominant” cluster, while both older age and male sex were associated with a higher likelihood of membership in the “Hypertension-Dominant” cluster. These findings empirically confirmed age and sex as key axes of cardiometabolic heterogeneity in this population and directly informed the subsequent stratified analyses.

Sex-stratified cluster analyses revealed three distinct phenotypes in both females (*n* = 225) and males (*n* = 211), with notable differences in metabolic patterns between sexes (Figure 3). Among females, Cluster 1 (“Young/Healthy,” *n* = 133, 59.1%) was the largest subgroup with a mean age of 50.0 years. This cluster exhibited minimal metabolic burden: hypertension 0.8%, type 2 diabetes 3.8%, dyslipidemia 10.5%, and central obesity 20.3%, the lowest prevalence for all metabolic conditions except central obesity. Cluster 2 (“Dyslipidemia-Dominant, Oldest,” *n* = 40, 17.8%) was the oldest female subgroup (mean age 70.4 years) and uniquely characterized by universal dyslipidemia (100%). This cluster also demonstrated high hypertension (72.5%) and moderate diabetes (25.0%) prevalence. Central obesity was present in 25.0%, the highest prevalence among female clusters. Cluster 3 (“Hypertension-Dominant,” *n* = 52, 23.1%) had a mean age of 67.0 years, similar to Cluster 2, but was distinguished by near-universal hypertension (88.5%) and complete absence of dyslipidemia (0%), a stark contrast to Cluster 2. This cluster had the highest diabetes prevalence among females (36.5%). Central obesity was present in 23.1%.

Among males, Cluster 3 (“Young/Healthy,” *n* = 117, 55.5%) was the largest subgroup with a mean age of 50.0 years and minimal metabolic burden across all conditions: hypertension 7.7%, type 2 diabetes 0.9%, dyslipidemia 10.3%, and central obesity 7.7%, notably lower than corresponding female values. Cluster 1 (“Oldest, Multi-Metabolic,” *n* = 49, 23.2%) was the oldest male subgroup (mean age 73.5 years), older than any female cluster. This cluster exhibited simultaneous elevation of multiple metabolic conditions: high hypertension (85.7%) and dyslipidemia (73.5%) with moderate diabetes (16.3%). Central obesity prevalence was 12.2%, the lowest among male clusters. Cluster 2 (“Diabetes-Driven, Middle-Aged,” *n* = 45, 21.3%) had a mean age of 62.8 years and was uniquely characterized by very high diabetes prevalence (75.6%), substantially higher than any female cluster. This cluster also showed near-universal hypertension (93.3%) but lower dyslipidemia (20.0%). Central obesity was present in 20.0%.

Both sexes exhibited large “Young/Healthy” clusters (females 59.1%, males 55.5%) with similar mean ages (50.0 years) and minimal metabolic burden. However, even within these healthy clusters, males had lower central obesity prevalence (7.7%) compared to females (20.3%). Among older clusters, females showed distinct single-metabolic-factor-dominant phenotypes: Cluster 2 with 100% dyslipidemia but 0% in Cluster 3, and Cluster 3 with 88.5% hypertension. In contrast, older male clusters exhibited co-occurring metabolic conditions: Cluster 1 combined high hypertension (85.7%) and dyslipidemia (73.5%), while Cluster 2 combined very high diabetes (75.6%) with near-universal hypertension (93.3%). We note the limited sample size in these clusters, which cautions us in interpreting the patterns observed here. 

T2DM prevalence patterns differed markedly between sexes. The highest female diabetes prevalence was 36.5% (Cluster 3), while males exhibited a diabetes-dominant phenotype with 75.6% prevalence (Cluster 2), more than double the highest female prevalence. Central obesity prevalence was consistently lower in males (7.7–20.0%) across all clusters compared to females (20.3–25.0%), despite similar or higher prevalence of other metabolic conditions in male clusters.

Age-stratified analyses revealed distinct phenotypes in participants younger and older than 65 years (Figure 4). Among those aged <65 years (*n* = 276), two clusters emerged. Cluster 1 (“<65, High-Risk Metabolic,” *n* = 66) was defined by universal hypertension (100%), high T2DM (39.4%), moderate central obesity (21.2%), and moderate dyslipidemia (27.3%), indicating early-onset multimorbidity. Cluster 2 (“<65, Metabolically Healthy,” *n* = 210) showed low prevalence across all conditions: central obesity 16.2%, T2DM 3.3%, dyslipidemia 13.8%, and no observed hypertension.

Among those aged ≥65 years (*n* = 160), three phenotypes were identified. Cluster 1 (“≥65, Healthy Older Adults,” *n* = 63) demonstrated a favorable metabolic profile despite older age, with low prevalence of central obesity (6.3%), T2DM (4.8%), hypertension (19.0%), and dyslipidemia (17.5%). Cluster 2 (“≥65, Hypertension- and T2DM-Dominant,” *n* = 43) was marked by near-universal hypertension (95.3%) and high T2DM (79.1%), with comparatively lower central obesity (25.6%) and dyslipidemia (18.6%). Cluster 3 (“≥65, Hypertension- and Dyslipidemia-Dominant,” *n* = 54) showed high hypertension (92.6%) and dyslipidemia (83.3%), but lower T2DM (13.0%) and central obesity (18.5%).

Taken together, age-stratified findings confirm that a subset of younger AAs with CHB already carries significant multimorbid risk, underscoring the importance of early cardiometabolic screening in this population. Among older adults, two distinct high-burden phenotypes, distinguished by whether dyslipidemia or T2DM co-occurs with hypertension, suggest that cardiometabolic risk evolves along divergent profiles with age. These phenotypic distinctions are hypothesis-generating and may inform future risk stratification and prevention strategies, pending prospective validation against clinical outcomes.

Furthermore, we conducted analyses to examine whether lifestyle behaviors, specifically, tobacco use and alcohol use, varied significantly across the clusters. Chi-square tests (available upon request) revealed no significant associations, indicating a similar level of health behavioral profiles across the clusters, in the overall sample and in the age- and sex-stratified clusters.

To descriptively characterize the sociodemographic profile of cluster membership, we performed multinomial logistic regression to examine how sociodemographic characteristics, including sex, education, annual income, nativity status, and age, were associated with class membership. The results (Supplementary Materials) showed that older age was associated with a higher likelihood of being in the “Dyslipidemia” cluster than in the “Young/Healthy” cluster, while being male and older were associated with a higher likelihood of being in the “Hypertension-Dominant” cluster than in the “Young/Healthy” cluster. Given that age was included as a clustering variable, these associations are interpreted descriptively rather than as independent predictors and further informed the decision to conduct age- and sex-stratified analyses.

## 4. Discussion

This study documents a substantial cardiometabolic burden among Asian Americans with CHB that extends well beyond what is captured by viral disease management alone. Nearly two-thirds of participants had at least one cardiometabolic component, and over one-quarter had two or more. Hypertension was the most prevalent condition (38.76%), followed by dyslipidemia (25.46%), T2DM (17.66%), and central obesity (16.74%). These findings are clinically significant because cardiometabolic conditions are not merely comorbidities in CHB but accelerants of fibrosis progression and hepatocellular carcinoma risk [35,36]. Clinical attention to the cardiometabolic comorbidities is needed for CHB management and cancer prevention.

The elevated cardiometabolic burden in this cohort is consistent with emerging evidence that CHB infection itself contributes to metabolic dysfunction through alterations in hepatic lipid handling, insulin signaling, and chronic inflammation [37,38,39]. The high dyslipidemia prevalence in our Dyslipidemia-Dominant cluster, comprising nearly one-fifth of the sample, is particularly notable, as lipid abnormalities in CHB are often under-recognized clinically [37,40]. Our findings suggest that routine lipid and metabolic screening may warrant consideration as a component of CHB care.

Cluster analysis revealed three phenotypically distinct exploratory groups that may have clinical relevance if validated prospectively. The largest cluster, “Young/Healthy”, may represent a potential prevention opportunity: a substantial proportion of this CHB population remains metabolically low-risk, and early targeted intervention warrants investigation as a means to potentially alter long-term trajectories. The two older clusters diverged sharply by condition profile: the “Dyslipidemia-Dominant” group exhibited universal dyslipidemia with high hypertension, while the “Hypertension-Dominant” group showed complete absence of dyslipidemia but carried the highest T2DM burden. This divergence in dyslipidemia prevalence between the two older clusters is an exploratory observation that illustrates the potential added value of phenotyping approaches in this population, though replication in larger samples is needed.

Sex-stratified analyses revealed meaningfully different metabolic aging profiles that were not apparent in the overall clustering solution. Among females, older clusters showed single-condition dominance, either near-universal dyslipidemia or near-universal hypertension, but not both simultaneously, although the limited size of these clusters cautions us in our interpretation of the patterns observed. Among males, older clusters showed co-occurring conditions; the oldest male cluster combined high hypertension and dyslipidemia, while the middle-aged cluster combined near-universal hypertension with a very high T2DM prevalence of 75.6%, more than double the highest female cluster. T2DM risk in AA women is frequently underestimated due to reliance on BMI-based screening thresholds that fail to capture metabolic risk at lower body weight [41,42,43]. Lower observed T2DM prevalence in female clusters should therefore not be interpreted as lower clinical urgency, as women with diabetes face disproportionately higher excess cardiovascular risk relative to men [44,45,46]. Additionally, chronic HBV infection has been associated with increased insulin resistance [47], and sex hormone differences in HBV immune response may partly explain divergent metabolic trajectories between sexes [48]. These divergent patterns suggest that sex-specific cardiometabolic trajectories may exist in this population, raising the hypothesis that screening approaches tailored by sex, such as intensified lipid and vascular risk assessment around the menopausal transition for women and earlier integrated metabolic monitoring for men, could be warranted.

This study has several limitations. First, hepatitis B classification relied on either EHR diagnosis or self-report, which may not definitively distinguish chronic from acute or resolved infection. Among participants with available EHR data, self-reported diagnosis showed high consistency with EHR-confirmed chronic hepatitis B, supporting the validity of our classification. However, this validation was limited to the subset of participants with linked EHR records, and misclassification cannot be excluded for remaining participants. Future studies should prioritize EHR-confirmed chronic hepatitis B classifications with supporting laboratory data (e.g., HBsAg positivity ≥6 months) to strengthen diagnostic precision. Second, we included age as a continuous clustering variable, which may have contributed to age-related separation between clusters. However, divergent metabolic profiles between the two similarly aged older clusters suggest that metabolic patterns drove differentiation beyond age alone. Formal cluster stability and internal validation indices (e.g., silhouette width, Dunn index) were not computed, which limits objective assessment of cluster quality. The consistency of phenotypes across overall, sex-stratified, and age-stratified analyses provides indirect evidence of solution stability. Third, we acknowledge that the extreme prevalence values observed in some clusters (e.g., 0% and 100% dyslipidemia) should be interpreted cautiously, as such patterns may partly reflect the tendency of clustering algorithms to create maximally differentiated groups when partitioning a limited number of binary variables, rather than representing fully distinct biological phenotypes. Future studies incorporating continuous cardiometabolic biomarkers such as systolic blood pressure, blood glucose, and waist circumference, rather than binary diagnostic classifications, may yield more nuanced phenotypic characterization. On top of that, the modest cluster sizes in stratified analyses may affect the stability and precision of cluster-specific prevalence estimates.

Another major limitation of this study is the substantial socioeconomic bias of the All of Us cohort, which limits generalizability to the broader Asian American CHB population. Our AA patient sample’s educational attainment and income level substantially exceed national estimates for AAs, which might be reflecting systematic underrepresentation of lower-SES Asian Americans in All of Us [49] and may have resulted in underestimation of true cardiometabolic burden. Findings should therefore be interpreted as characterizing cardiometabolic phenotypes among a relatively educated, higher-SES AA sample rather than AAs with CHB broadly. Moreover, the All of Us dataset combines survey responses, physical measurements, and EHR diagnoses collected at different time points without a defined synchronization window, which may have introduced temporal discordance in cardiometabolic condition classification and should be considered when interpreting cluster assignments. On top of that, we note that the cross-sectional design precludes causal inference, and temporal relationships between CHB activity and cardiometabolic disease onset cannot be established. Although the All of Us dataset includes Asian ethnic subgroup data, sample size constraints precluded disaggregated analyses across East Asian, South Asian, and Southeast Asian communities, which is a limitation given known metabolic heterogeneity across these groups [50,51,52]. Future longitudinal studies integrating viral biomarkers, inflammatory markers, and imaging-based liver fat quantification are needed to clarify mechanistic pathways and evaluate whether aggressive cardiometabolic risk modification improves hepatic and cardiovascular outcomes in CHB populations.

We also note one methodological limitation: the absence of a definitive elbow in within-cluster sum-of-squares plots introduced some subjectivity into the cluster selection process. Lastly, we acknowledge that a small proportion of central obesity classifications (10 of 73, 13.7%) relied on self-reported obesity as a proxy for missing waist circumference data; given the limited number of affected cases, this is unlikely to have meaningfully influenced prevalence estimates or cluster assignments.

While no significant associations were found between cluster membership and lifestyle behaviors, two important caveats merit consideration. First, the prevalence of tobacco use (4.59%) and frequent alcohol consumption (10.32%) was notably low in our sample, which may limit statistical power to detect behavioral differences across clusters, particularly in stratified analyses with smaller cell sizes. However, it is important to note that tobacco and alcohol use are well-documented health concerns in Asian American communities [53,54,55], with some subgroups showing markedly higher prevalence than our estimates. The low behavioral prevalence in our sample likely reflects recruitment bias inherent to the All of Us cohort, which overrepresents high-socioeconomic-status individuals with college education, rather than true population-level rates. Future studies should recruit more socioeconomically diverse AA samples to adequately examine behavioral-metabolic phenotype relationships.

## Figures and Tables

**Figure 1:**
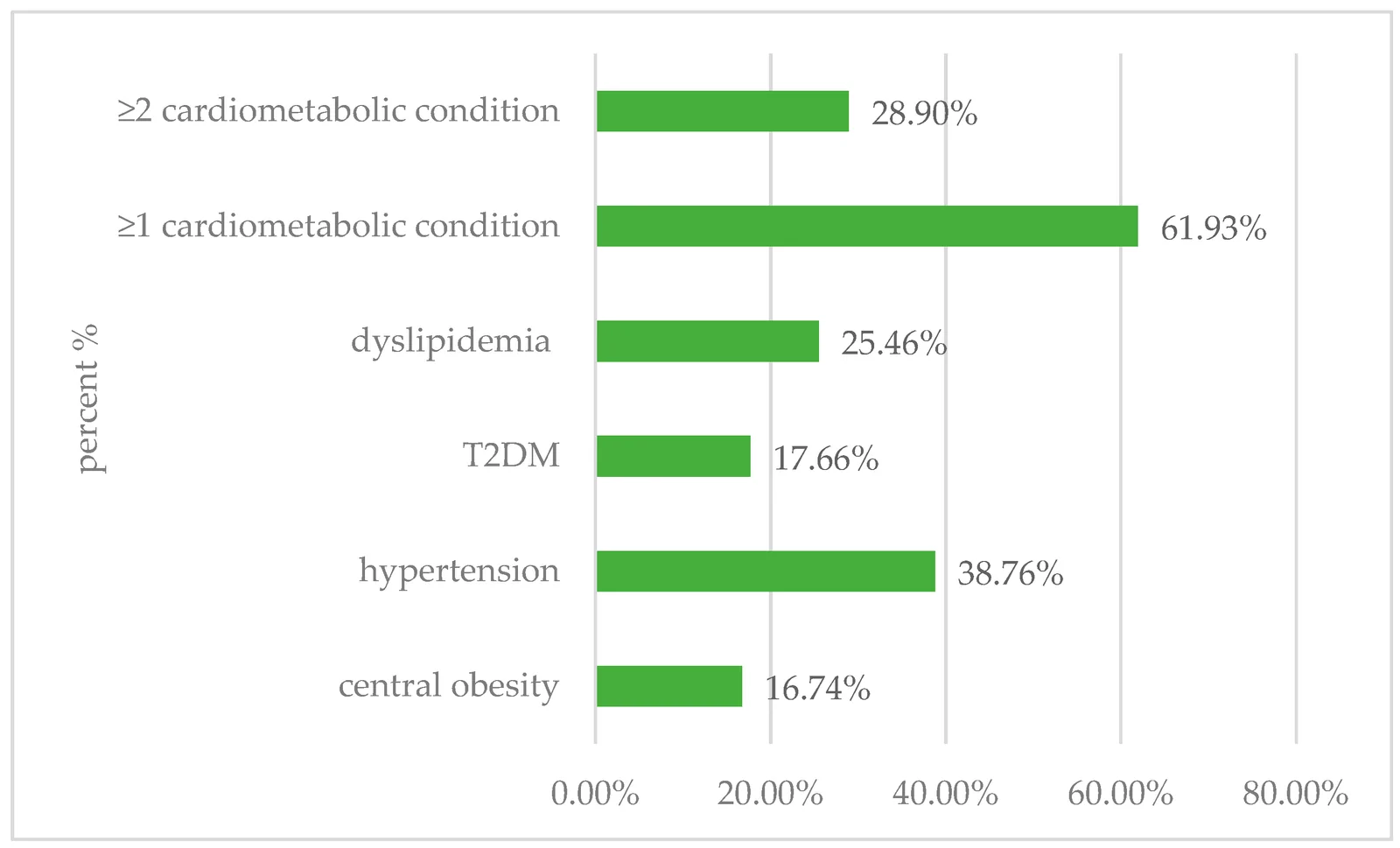
Prevalence of cardiometabolic conditions in Asian Americans with chronic hepatitis B.

**Figure 2:**
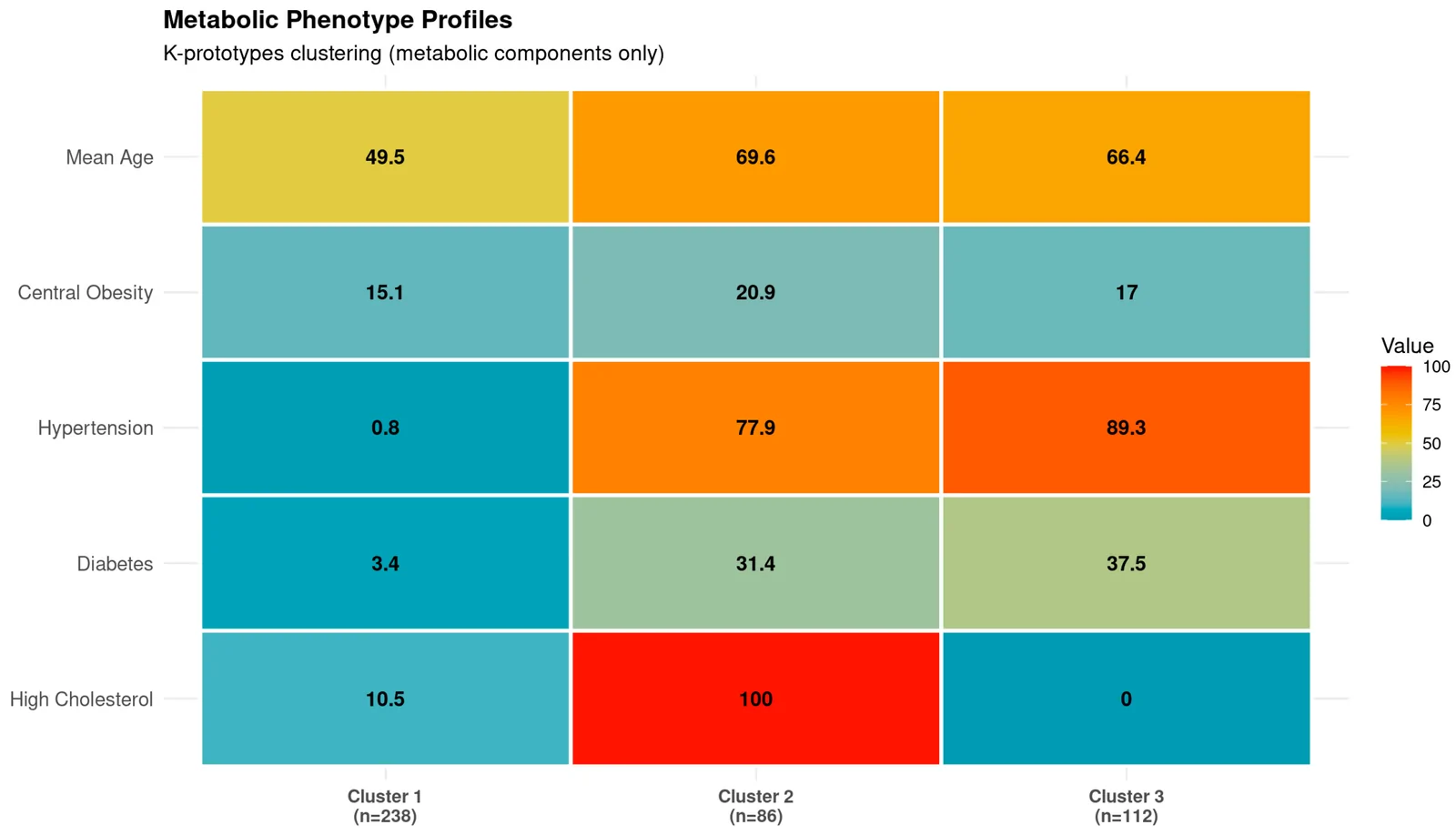
Metabolic phenotype profiles in Asian Americans with chronic hepatitis B.

**Figure 3:**
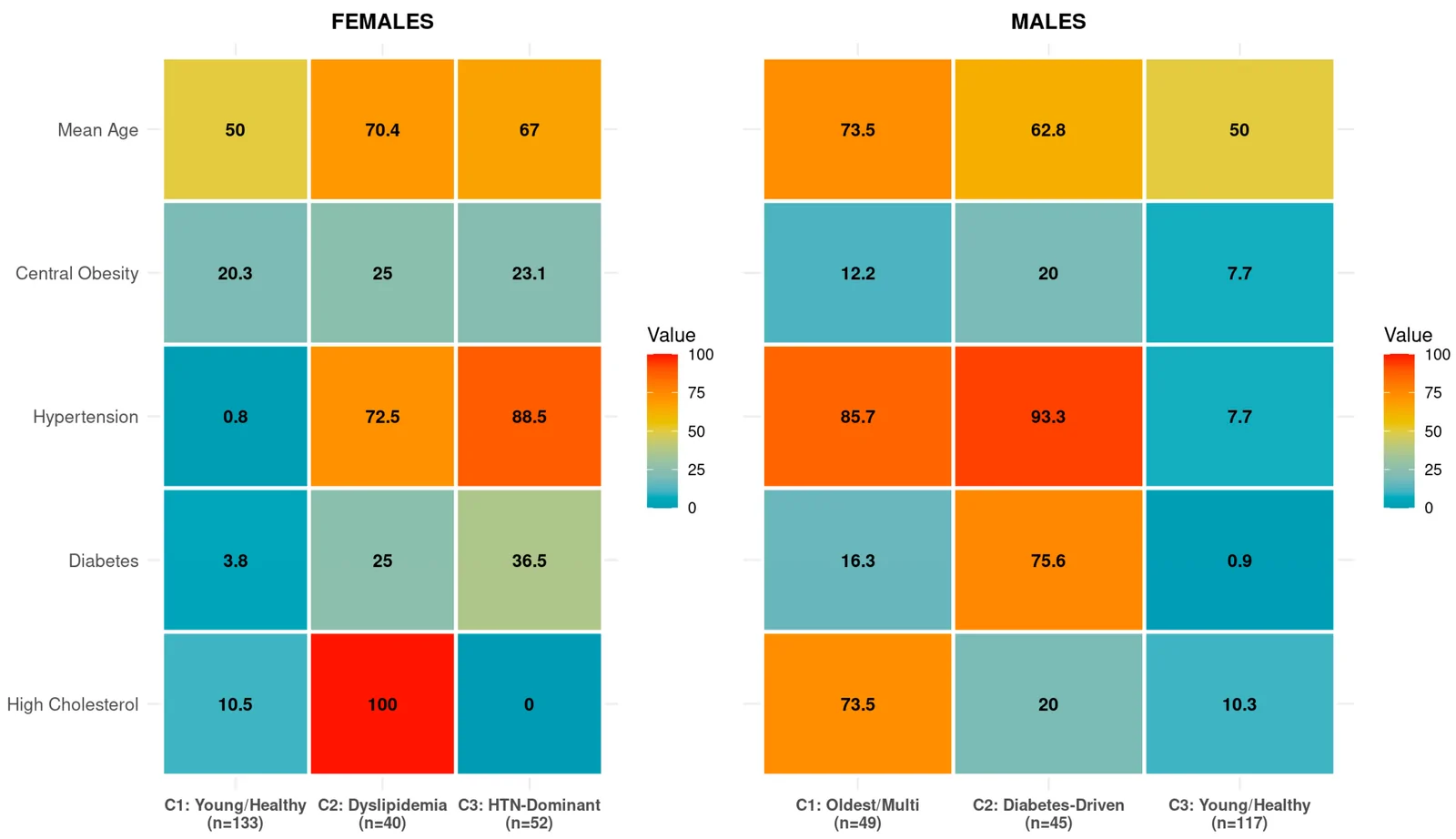
Metabolic phenotype profiles in Asian Americans with chronic hepatitis B, stratified by sex.

**Figure 4:**
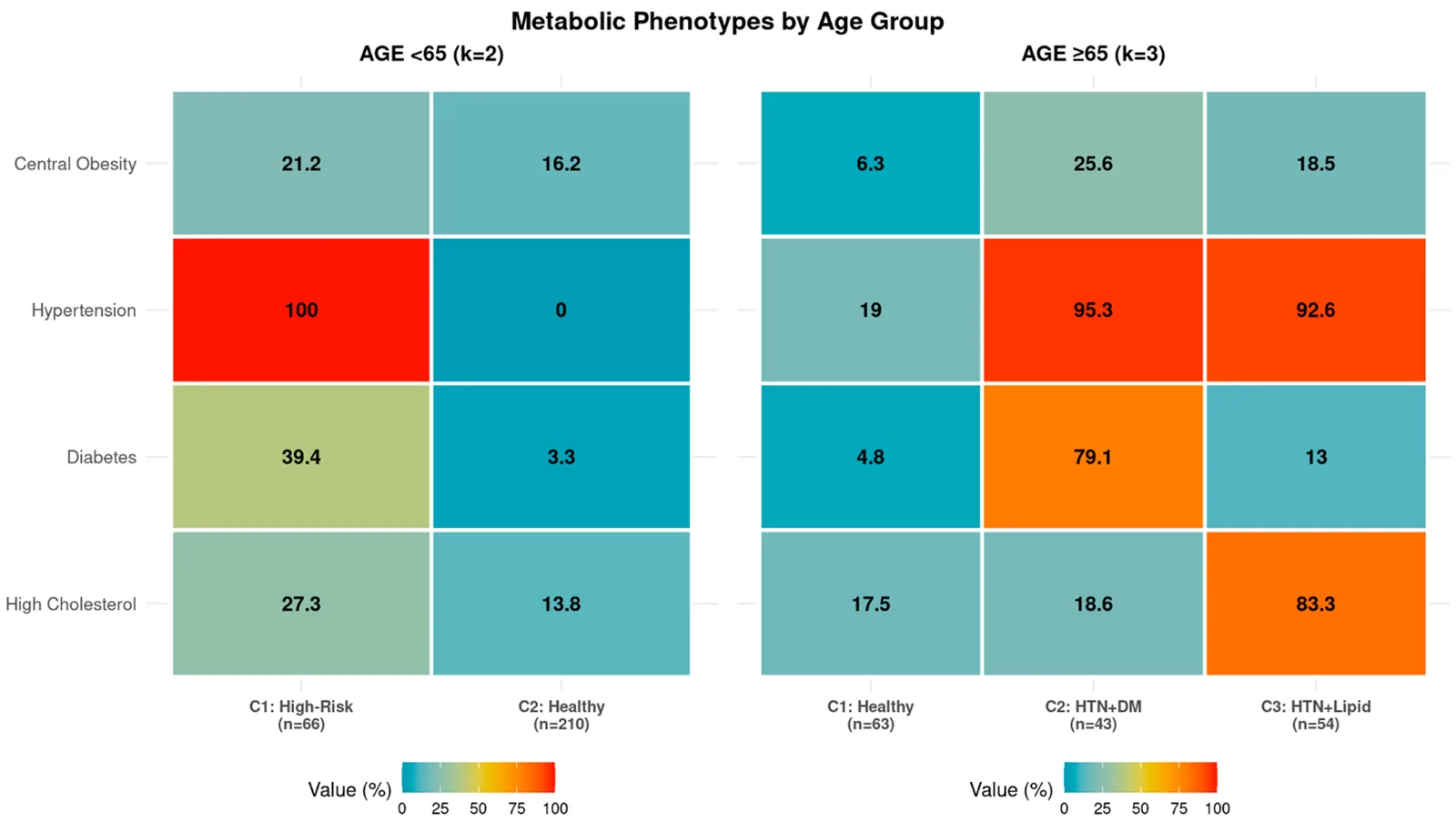
Metabolic phenotype profiles in Asian Americans with chronic hepatitis B, stratified by age group.

**Table 1:** Sociodemographic characteristics of the study population: Asian Americans with chronic hepatitis B (N = 436).

Characteristic	*n* (%)
Sex	
Female	225 (51.61)
Male	211 (48.39)
Age Group (years)	
18–44	91 (20.87)
45–64	185 (42.43)
≥65	160 (36.70)
Marital Status	
Married/Domestic Partner	305 (69.95)
Separated/Divorced/Widowed	131 (30.05)
Nativity	
Foreign-born	359 (82.34)
U.S.-born	77 (17.66)
Education	
College or above	388 (88.99)
Less than college	48 (11.01)
Annual Household Income	
<$50,000	152 (34.86)
≥$50,000	284 (65.14)
Tobacco Use	
No	416 (95.41)
Yes	20 (4.59)
Alcohol Use	
Not a frequent drinker	391 (89.68)
Frequent drinker	45 (10.32)

## Data Availability

Data used in this study can be accessed at the All of Us data website: https://www.nih.gov/allofus/research (accessed on 20 September 2026).

## Supplementary Materials

The following supporting information can be downloaded at: https://www.mdpi.com/article/10.3390/jcm15197371/s1, Table S1. Multinomial Logistic Regression: Predictors of Metabolic Phenotype Membership.

## Abbreviations

The following abbreviations are used in this manuscript:

AA	Asian American
AoU	All of Us
BMI	Body Mass Index
CHB	Chronic Hepatitis B
DV	Direct Volunteer
EHRs	Electronic Health Records
HCC	Hepatocellular Carcinoma
HIV	Human Immunodeficiency Virus
HPO	Health Care Provider Organization
ICD	International Classification of Diseases
MASLD	Metabolic Dysfunction-Associated Steatotic Liver Disease
MetS	Metabolic Syndrome
T2DM	Type 2 Diabetes Mellitus
