# Supplementary material for: A Multidimensional Assessment of Cardiometabolic Risk in Asian Americans with Chronic Hepatitis B Using the All of Us Datasets
Source: J Clin Med. 2026 Sep 23;15(19):7371. doi: 10.3390/jcm15197371 (PMC13665728; doi:10.3390/jcm15197371)
Supplement: Supplementary file 1 [file jcm-15-07371-s001.zip › jcm-4547575-supplementary.pdf]

Supplementary Table S1. Multinomial Logistic Regression: Predictors of Metabolic Phenotype Membership.

	**OR**[1]	**95% CI**	**p-value**
**Dyslipidemia**			
Sex			
Female	—	—	
Male	1.43	0.78, 2.64	0.3
Education			
College or above	—	—	
Less than college	0.31	0.08, 1.12	0.074
Annual Income			
< 50k	—	—	
≥ 50k	0.79	0.40, 1.57	0.5
Nativity Status			
Foreign born	—	—	
US born	0.97	0.43, 2.19	>0.9
Age (years)	1.16	1.12, 1.20	**<0.001**
**HTN-Dominant**			
Sex			
Female	—	—	
Male	1.74	1.01, 3.02	**0.047**
Education			
College or above	—	—	
Less than college	2.13	0.90, 5.04	0.087
Annual Income			
<50k	—	—	
≥50k	0.85	0.45, 1.60	0.6
Nativity Status			
Foreign born	—	—	
US born	0.84	0.39, 1.78	0.6
Age (years)	1.12	1.09, 1.15	**<0.001**

[1] OR = Odds Ratio; Reference group: Young/Healthy cluster

Abbreviations: CI = Confidence Interval, OR = Odds Ratio
